# Bright field microscopic cells counting method for BEVS using nonlinear convergence index sliding band filter

**DOI:** 10.1186/1475-925X-13-147

**Published:** 2014-10-24

**Authors:** Dong Sui, Kuanquan Wang, Heemin Park, Jinseok Chae

**Affiliations:** Biocomputing Research Center, School of Computer Science and Technology, Harbin Institute of Technology, Harbin, China; Department of Computer Software Engineering, Sangmyung University, Cheonan, Korea; Department of Computer Science and Engineering, Incheon National University, Incheon, Korea

**Keywords:** Cell counting, Transformed sliding band filter, BEVS, Microscopy image processing

## Abstract

**Background:**

The Baculovirus Expression Vector System (BEVS) is a very popular expression vector system in gene engineering. An effective host cell line cultivation protocol can facilitate the baculovirus preparation and following experiments. However, the counting of the number of host cells in the protocol is usually performed by manual observation with microscopy, which is time consuming and labor intensive work, and prone to errors for one person or between different individuals. This study aims at giving a bright field insect cells counting protocol to help improve the efficient of BEVS.

**Method:**

To develop a reliable and accurate counting method for the host cells in the bright field, such as Sf9 insect cells, a novel method based on a nonlinear Transformed Sliding Band Filter (TSBF) was proposed. And 3 collaborators counted cells at the same time to produce the ground truth for evaluation. The performance of TSBF method was evaluated with the image datasets of Sf9 insect cells according to the different periods of cell cultivation on the cell density, error rate and growth curve.

**Results:**

The average error rate of our TSBF method is 2.21% on average, ranging from 0.89% to 3.97%, which exhibited an excellent performance with its high accuracy in lower error rate compared with traditional methods and manual counting. And the growth curve was much the manual method well.

**Conclusion:**

Results suggest the proposed TSBF method can detect insect cells with low error rate, and it is suitable for the counting task in BEVS to take the place of manual counting by humans. Growth curve results can reflect the cells’ growth manner, which was generated by our proposed TSBF method in this paper can reflected the similar manner with it’s from the manual method. All of these proven that the proposed insect cell counting method can clearly improve the efficiency of BEVS.

## Background

### Biology background

The baculoviruses are double-stranded DNA viruses containing a large genome up to 180,000 bp, and are arthropod specific viruses with two different phenotypes during different infection stages [1, 2] including budded viruses (BVs) and occlusion-derived viruses (ODVs). Depending on the distinct structural and biological characteristics, the baculoviridae family can be divided into four genera: α-baculovirus, β-baculovirus, γ-baculovirus, and δ-baculovirus [2, 3]. Traditionally, these kinds of arthropod specific viruses are employed to control insect pests in soybean fields, cotton bollworm and helicoverpa armigera. Very recently, with the development of microbiology and virology, the special properties of high level expression for very late genes make them suitable vectors for the delivery of foreign genes. So far, Autographa California Multiple Nucleopolyhedrovirus (AcMNPV) and lesser extent Bombyxmori Nucleopolyhedrovirus (BMNPV) are broadly applied as alien gene vectors to produce recombinant proteins in many hosts [2, 3].

The baculovirus expression vector system (BEVS) has been widely used to express heterologous genes in various cell lines since the mid 1980’s [4]. With recent advances in cell culture and molecular manipulations, many special media, transfection reagents and expression vectors have been developed for applications. As an excellent expression system, BEVS contains efficient promoters that can provide ideal production of the recombinant proteins. Its host insect cell lines, serum-supplemented or serum-free growth media and infection strategies also allow optimal virus production and gene expression. In addition, the scalable process for the culture of insect cell lines makes the downstream processing more convenient in labs. Together, these advantages enable its large-scale applications in gene expression and protein production. Furthermore, the broad range of susceptible cell lines and the nature in cells entrancing without toxic and replication make baculovirus an excellent tool for studying the expression and function of genes, which allows the BEVS to be successfully applied to gene therapy, pharmaceutical and vaccine productions.

**Figure 1:**
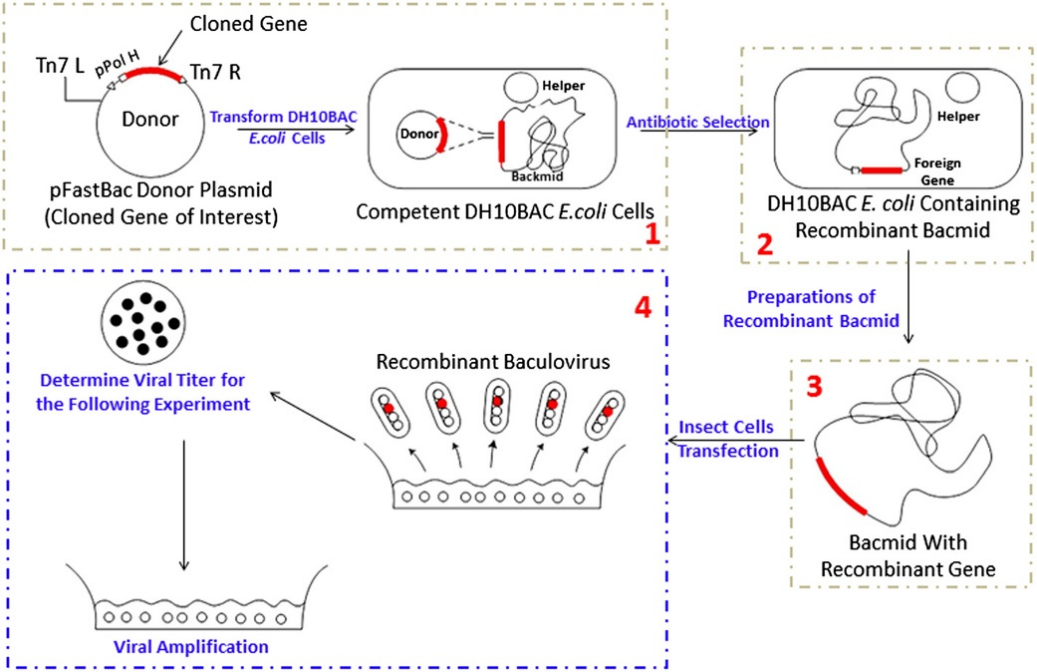
**Recombinant baculoviruses and gene expression protocol using the bac-to-bac expression system; step 1.** Construction of donor plasmid; step 2. Production of bacmid; step 3. Recombinant bacmid preparation; step 4. Production of recombinant baculovirus.

Figure 1 explains the classical protocol of recombinant-baculoviruses production and gene expression using the Bac-to-Bac Expression System. Firstly, a recombinant donor plasmid is constructed, and then the plasmid was transformed into DH10BAC *E.coli* competent cells (step 1 in Figure 1) to produce recombinant Bacmid through homologous recombination (step 2 in Figure 1). After preparing of recombinant Bacmid (step 3 in Figure 1), the host insect cells, are transfected by the extracted Bacmid. Finally, the recombinant baculovirus containing a cloned gene is prepared from the product of insect cell disruptions (step 4 in Figure 1).

As Figure 1 shows, the hosts, such as *Sf9* insect cells, are critical for producing the recombinant baculovirus and the insect cells’ density (1 × 10^6^-2 × 10^6^ cells/ml) are very important for the follow-up experiments. An effective culture protocol of insect cells can facilitate the virus preparation. In spite of its key rolls, the counting of insect cells usually takes lots of time and is labor intensive by traditional methods in lab because it is usually manipulated by humans under microscopy. Moreover, traditional methods are even prone to cause errors without being repeated by different people. It should be noted that there are still no efficient computer-aided methods to solve these problems in regard to the BEVS protocol. In this paper, we propose a bright field insect cell counting method based on the Nonlinear Convergence Index Sliding Band Filter to improve the protocol efficiency.

### Related works

Cell counting is an indispensable and essential problem because it directly affects the efficiency of many cell-based gene expression systems like BVES. Traditionally, this task is usually performed by microscopic-based counting. For example, the Neubauer, Burker and Fuchs-Rosenthal chambers are well known methods for counting cells in different cell concentration of interest [5]. However, all of these methods have to be manually manipulated and therefore are prone to cause errors for the same person or different persons. Furthermore, most of them require frequent repetitions for validations [6]. In the 1940s, Wallace Coulter introduced a suspended particles counting method in a fluid to provide an automatic cell counting tool without lab worker dependencies, which is a milestone in solving cell counting automatically [5]. Following this milestone, automated human blood counting tools based on microscopic image analyses with high performance became commercially available. However, there are still many defects to be improved [7–9]. All of these defects should be addressed in order to develop automatic cell counting and analysis tools to facilitate the cell based experiments. In this paper, we focus on an image processing based insect cell counting method for BVES. To the best of our knowledge, there are no existing methods that have the same purpose with our proposed method. Beyond that, the proposed method can also be applied to other kinds of cells under microscopy with a bright field.

As a basic problem of computer-aided biology experiments, microscopic cell image analysis in a dark field has been explored by many researchers. Image processing based cell counting methods have been widely used for cell-based biological experiments during the past decades, especially for red blood cells and white blood cells [10]. Detecting and counting cells in microscopy images for biological systems is a key factor that affects system efficiency. Traditionally, classical computer-aided methods solve this problem by applying image segmentation, which is a fundamental but difficult problem in computer vision. These methods mainly focus on cell images with high contrast between cells and their background; this can be described as the dark field cell identification problem. Several image filters and segmentation methods were employed for cell identification and counting in microscopy images, such as, watershed transform-based methods [11], morphological operators-based methods [12], gray level threshold based methods [13], contour or region-based methods [14], minimum-error-threshold histogram-based methods [14], and Artificial neural network (ANN) was also used for investigating the same problem [14]. On the other hand, various commercial or free software have been developed for solving cell counting in different image datasets, such as ImageJ and Cellprofiler [7]. However, most of these methods and software mainly focused on processing some types of cells or cell lines and cannot be applied to other cell types. Another problem regarding these methods is that they are either semi-automated or require re-adjusting parameters on the same images to obtain accurate results. Additionally, the aforementioned methods and commercial software cannot obtain accurate results without human interventions, and some methods must be manipulated by experienced biology technical staffs that are trained in digital image processing. It can draw a conclusion that more robust and reliable tools are needed for the biotechnologist in larger cell image data sets. Furthermore, another challenge we have faced is the bright field cell counting which has had rare attentions so far.

In this paper, we propose an effective cell counting method for the insect-baculovirus expression system using Transformed Sliding Band Filter (TSBF), which is a new image-based microscopy cell counting method under a bright field. The TSBF method is based on a nonlinear filter, Sliding Band Filter (SBF), which was proposed for lung nodule detection and dark field fluorescein stained cell nuclei identification [15–17], and was also employed for density packed cell counting in another work of our group. This filter is based on gradient vector convergence which is capable of detecting low contrast cell nuclei and cytoplasm information lost in the background noise [15]. It can also reduce the uncertainty caused by noise. Furthermore, the parameters of the filter are directly related to cell shape and size, which leads to easy and intuitive setup by the biotechnologist who knows little about image processing techniques. In our method, we evaluated the gradient vector convergence in the bright field, and transformed the convergence index in the SBF filter to fit the location we want to detect. The results show that the TSBF method can be applied to insect cells in different infecting stages, and can improve the efficiency in the BEVS protocol.

## Methods

### Workflow of our proposed pipeline

**Figure 2:**
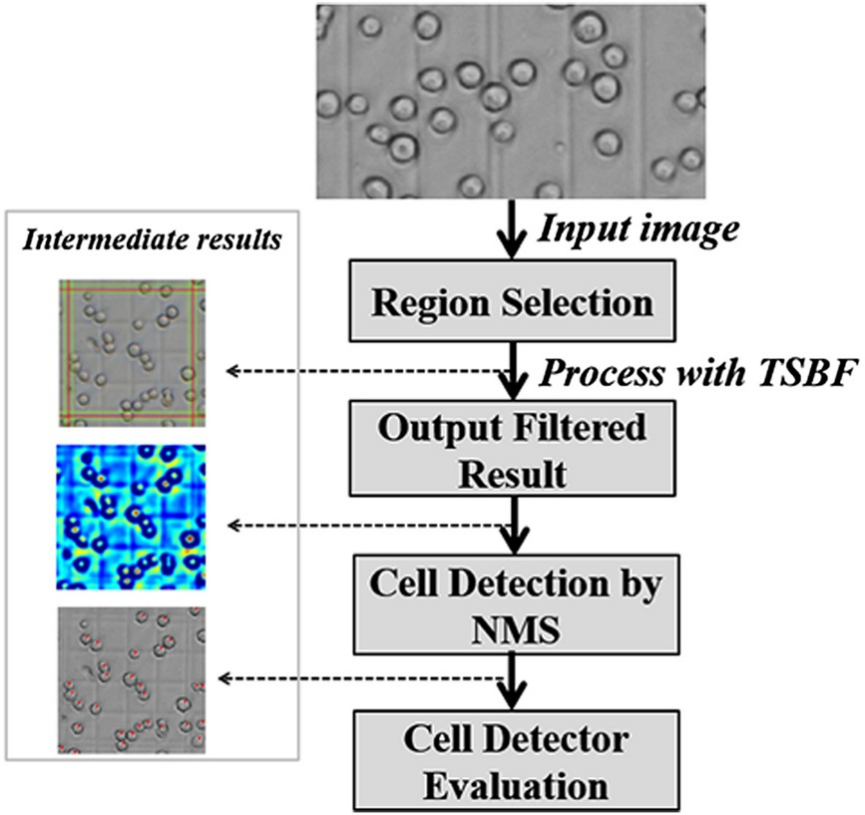
**Cell detection diagram, using example images from the Sf9 insect cells dataset illustrating our approach.**

Firstly, after collecting the image dataset, images of region A1 through A5 in Figure 2 were selected manually as shown in Figure 2. Our approach to insect cell counting in bright field microscopy images is based on local TSBF image filtering. The distributions of gradient vectors for cells in bright and dark fields were compared, and then the SBF filter was transformed according to the gradient vectors in the bright field to make it match the cell gradient vector distribution manner well. Like most work on cell identification and counting, our approach conducts cell image enhancement as an initial step. Given the enhanced images by TSBF, a non-maximal suppression filter was applied to search local maxima. Subsequently, cell centers were tentatively associated with the locations of maxima responses.

After cell detection, the accuracy of the TSBF method was analyzed and compared with the manual method, and this yields the final result of our approach. In this section, the characteristics for cell images in both dark and bright fields are introduced. Finally, an evaluation method is introduced for comparing with manual counting and some computer-aided approaches.

### Host cell preparation

As the host of baculovirus, there are four types of insect cell lines commonly used for BVES applications as listed in Table 1. These insect cell lines support various levels of expression and differential glycosylation with the same recombinant protein [18]. We choose the most widely used cell type, *Sf9*, a clonal isolated from the *Spodoptera frugiperda* cell line *IPLB-Sf21-AE*, as the host cells, which was support by Harbin Veterinary Research Institute, CAAS.

**Table 1:** **Commonly used insect cell lines in BVES**

Insect species	Cell line
*Spodoptera frugiperda*	Sf9
*Spodoptera frugiperda*	Sf-21
*Trichoplusia ni*	Tn-368
*Trichoplusia ni*	High-Five™ BTI-TN-5B1-4

After recovering from liquid nitrogen, *Sf9* insect cells were diluted by SF900 II complete medium (Cat. No. 10902-088, Invitrogen Inc., USA) with 10% fetal bovine serum (Cat. No. A15-043, PAA Inc., Austria) and 1% double-antibiotic (Penicillin and Streptomycin, Takara Inc., Japanese). Cell suspension with a density of 5 × 10^5^ cells/mL was prepared at 27°C for the counting task in different concentrations. After the density reaches 2 ~ 3 × 10^6^ cells/mL, the population of the insect cells grow exponentially and various kinds of experiments were conducted during this period. When its density reaches 5 ~ 6 × 10^6^ cells/mL, it comes into the stationary phase with no growth of cells and descends.

### Manual counting data collection

Insect cell densities in a suspension culture medium are usually calculated by a Hemacytometer, because it includes enough regions to evaluate both low and high concentration of insect cells [5], see Figure 2 for details.

Traditionally, the insect cells are diluted at a certain ratio using Sf900 II medium according to the period of cell cultivation. Then, they are injected into the chamber where the volume (the depth and area) is standardized. The cell number *Squire*_*In* sec *t* _ *cell*_ from the field was obtained by the visual inspection in 1 mm^2^ regions in the chamber. Finally, the density of insect cells *Vol*_*INC*_ is calculated by Eq. 1.
1

Where *C*_*depth*_ is the depth of the chamber and *Cell*_*cct*_ is the concentration of the insect cells.

**Figure 3:**
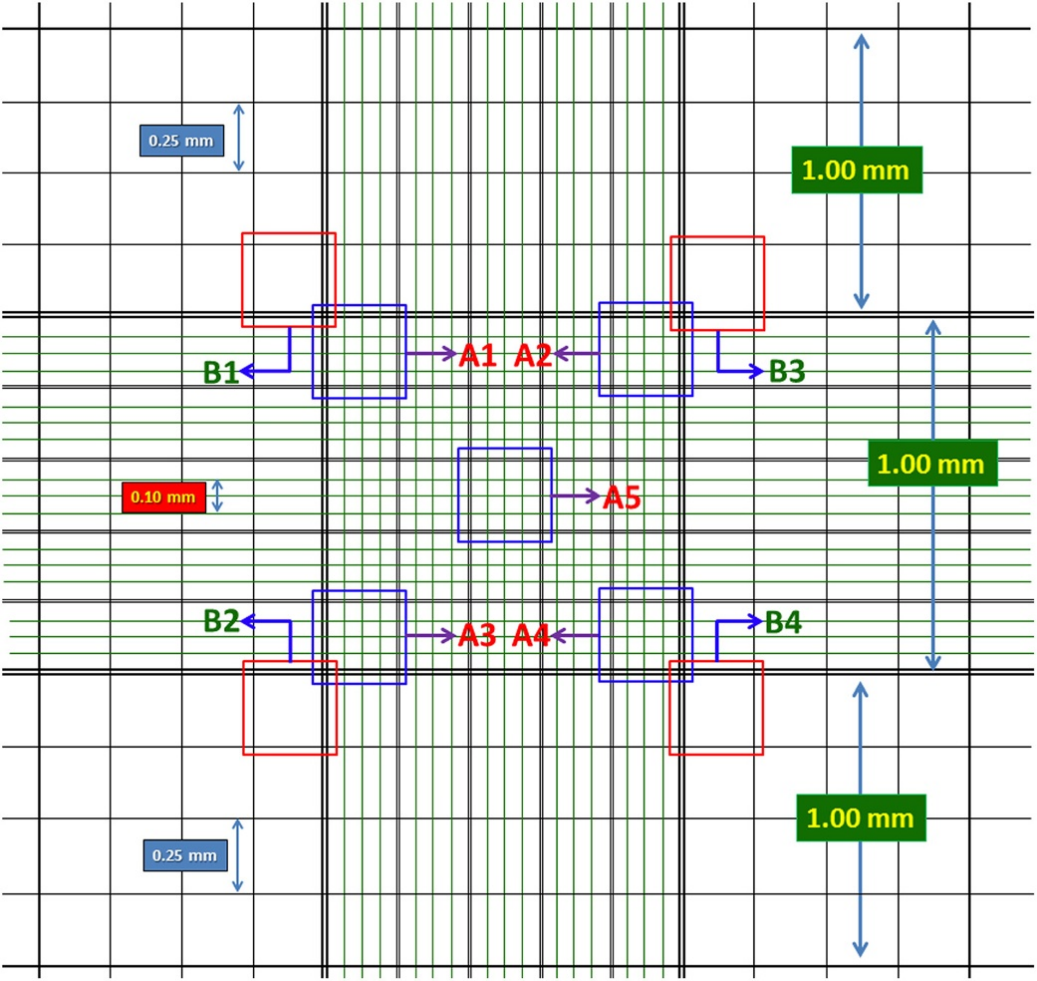
**Scheme of Hemacytometer with nine 1 × 1 mm** ^**2**^ **squares with 0.1 mm depth.** The A1 toA5 region is divided into 1/25 mm^2^ regions, and the B1 to B4 region is divided into1/16 mm^2^ regions.

Typically, visual evaluation for insect cells is calculated from 4 or 5 regions. An example of the 4 regions and 5 regions are B_1_, B_2_, B_3_, B_4_ and A_1_, A_2_, A_3_, A_4_, A_5_, respectively, as shown in Figure 3. In this paper, we mainly focus on region A_1_ through A_5_.

### Image data set acquisition

*Sf9* insect cell images were collected using optical model of Nikon Te2000 confocal microscopy with 20 × 10 (objective × eyepiece) for finding counting region and 40 × 10 (objective × eyepiece) for performing the cell counting task in BEVS. For each 24 hours until cells grow into the stationary phase, 3 samples were collected separately and marked as sample 1, sample 2 and sample 3 according to the collected sequence. Microscopic images of region A1 through A5 were collected separately from each sample.

### Differences of cell images in dark and bright field

**Figure 4:**
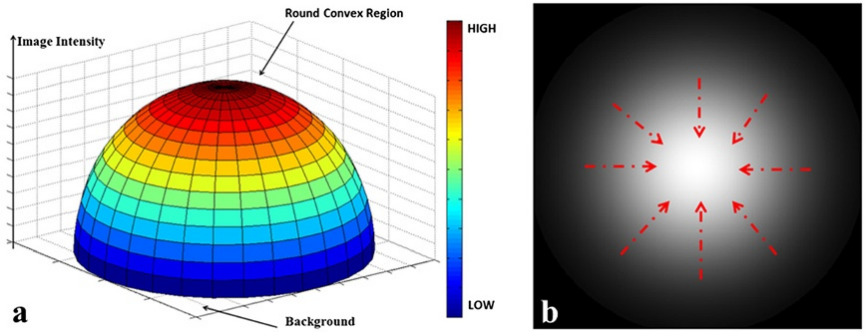
**Scheme of rounded convex region. (a)** Map of Round convex region with image intensity; **(b)** Distribution of Gradient Vectors in the Convex Region.

Traditionally, the cells in the dark field appear to have a higher intensity in the center than the edge, and this can be described as a rounded convex region [15]. This region is defined as: the region’s equi-contours of intensity are concentric and all gradient vectors in the area toward the center. Figure 4(a) presents the round convex region related to image intensity considered in this paper. Figure 4(b) shows a typical round convex region image in a dark background Red arrows indicated the convergence directions of gradient vectors.

**Figure 5:**
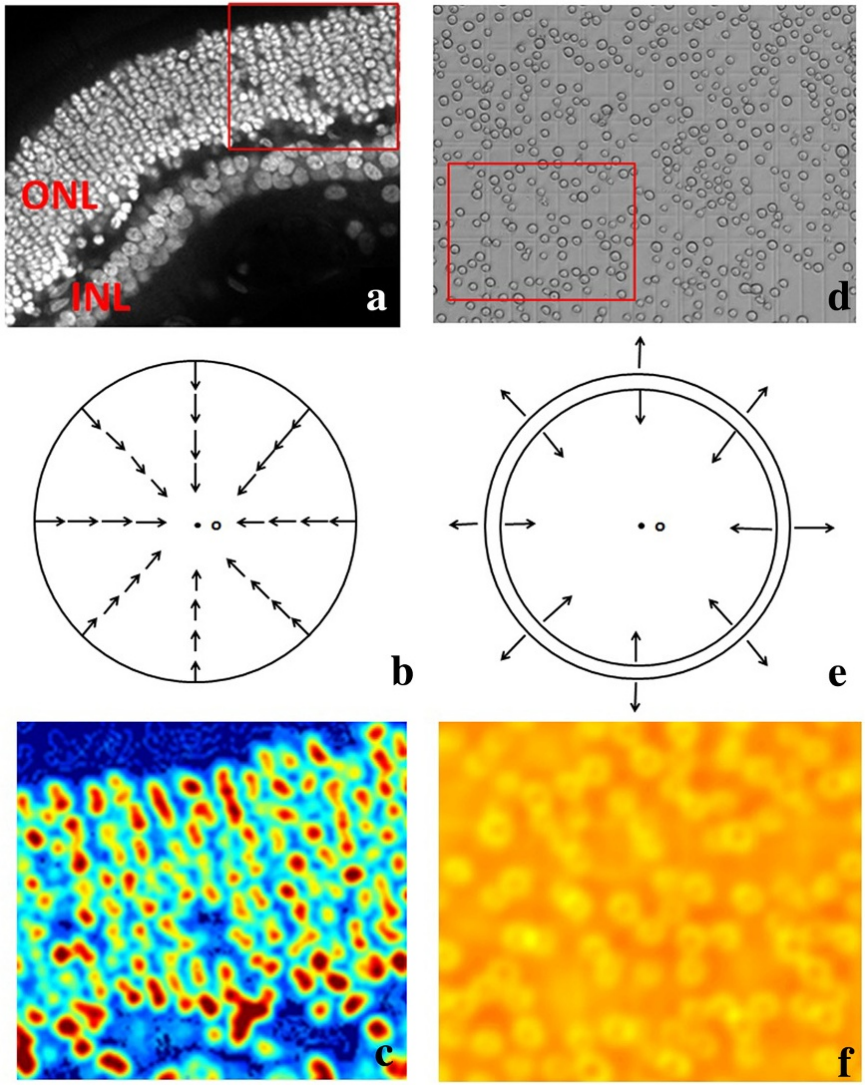
**Comparison of gradient vector distribution and SBF filter enhancement results between the dark field and bright field. (a)** Retinal cells image in the dark field. **(b)** Distribution of gradient vector for each retinal cell in **(a)**. **(c)** Result of SBF filter enhancement on **(a)**. **(d)** Sf9 insect cells in the bright field. **(e)** Distribution of gradient vector for each insect cell in **(d)**. **(f)** Result of SBF filter enhancement on **(d)**.

Cells in single plane dark field images always exhibit individual nuclei with discontinuities in the boundary. As it is widely known, the distribution of genetics materials (such as DNA) always concentrates at the center of the cell nuclei area in a living cell, which is related to the image intensity after staining by fluorescence dyes. Figure 5(a) shows a single plane micrograph in both the ONL (Outer Nuclear Layer) and INL (Inner Nuclear Layer) of human retinal. http://www.bioimage.ucsb.edu/image-processing/retina.

The retinal ONL cells were stained by nuclei dye, TO-PRO (excitation wavelength 642 nm, emission wavelength 661 nm). Image data sets was published in [19]. The retinal cell DNA concentrated at the center of each cell leading to the intensity of cell nuclei appears much higher than the edge in the single plane cell image. Thus, the individual cell in the dark field can be simulated as simple, nearly circular shapes, which refers to a rounded convex region. Figure 5(b) presents the gradient vector distribution of each cell. In addition, after being enhanced by the SBF filter, as depicted in Figure 5(c), the center was enhanced.

**Figure 6:**
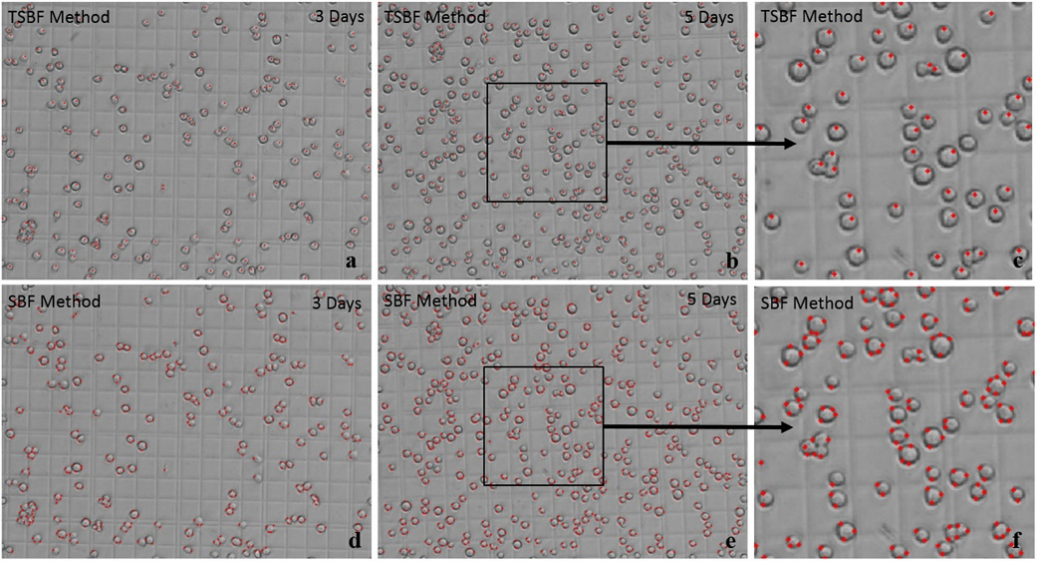
**Sf9 insect cell detection comparison between TSBF and SBF method. (a)** TSBF detecting result on the 3^rd^ day. **(b)** TSBF detecting result on the 5^th^ day. **(c)** Magnification of part of **(b)**. **(d)** SBF detecting result on the 3^rd^ day. **(e)** SBF detecting result on the 5^th^ day. **(f)** Magnification of part of **(e)**.

Unlike the dark field, insect cells in the bright field usually shows the whole living cell body and the cytoplasm is not visible compared with the cell membrane. Figure 5(d) shows the insect cells in the bright field compared with the retinal cells in the dark field (Figure 5(a)). The boundaries of an insect cell in the bright field shows a clearly boundaries of cytoplasm and the edge exhibited discontinuity between inside and outside of the cell. The gradient vector around the cell membrane area shows convergence toward the cytoplasm center and divergence away from the cell membrane toward the background, and this was modeled as a transformed rounded convex region enhanced by our proposed method discussed in part B. Figure 5(e) indicated the gradient vector distribution inside and outside of the insect cells. Unfortunately, as shown in Figure 5(f), the SBF filter does not work in the bright field, and it can only detect the region of the cell membrane instead. Figure 6(a) to (f) exhibited the detection results of comparison between the TSBF and SBF method in the 3^rd^ and 5^th^ day. TSBF can detect the center of each cell as one, but the SBF can only detect the cell membrane region and it detect one cell as two, three or even more cells.

### Cell detector design using TSBF

Cell detection is an important task in cell counting and recognition, which is usually achieved by intensity threshold or image segmentation [20]. However, these methods assume that cells are isolated from each other in a dark field [21, 22]. In this paper, insect cells in images appeared as ring structures with different intensities. The most challenging problem in cell detection is that images in this case are obtained by bright field microscopy. In addition, most of the aforementioned approaches require ideal environments that do not have irregular illumination, channel cross talk and noise caused by the microscopy [20].

In this paper, a new method for insect cells counting is proposed, which uses image filtering to enhance certain features on gray level images [23]. Considering most of the linear filters have small support regions, i.e., m × m pixels where *m* ∈ {2, 3, 5......}, in this paper, a Transformed Sliding Band Filter with larger support regions is employed to enhance insect cells in a bright field. TSBF was originated from a nonlinear filter, the Sliding Band Filter (SBF), a member of the Convergence Index (CI) family.

The CI family was design for enhancing the rounded convex region in digital images, and was based on the maximization of CI at each pixel of spatial coordinates (*x*, *y*). The Convergence Index is defined by the following formulations:
2

where (*x*, *y*) is the coordinate of the interesting pixel, *Pn* is the number of pixels in the support region *R*, *α*(*m*_*R*_, *n*_*R*_) is the angle between the gradient vector computed at pixel (*m*_*R*_, *n*_*R*_) and the line connected (*x*, *y*) and (*m*_*R*_, *n*_*R*_).

**Figure 7:**
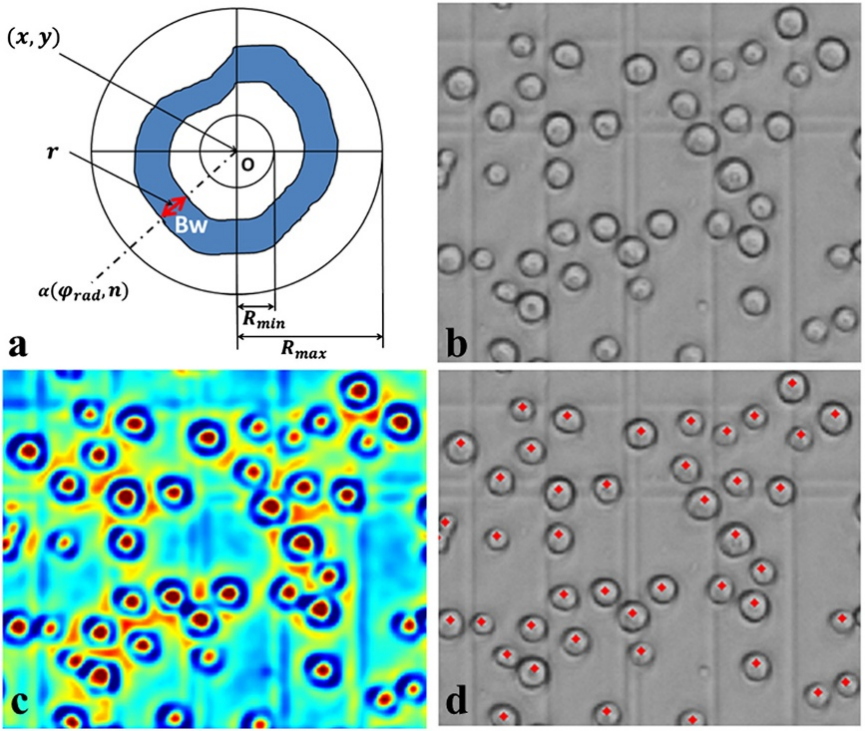
**TSBF for cell detection. (a)** Schematic of the SBF supports region; **(b)** *Sf9* insect cells under microscopy; **(c)** image filtered by TSBF; **(d)** cell detected results on the same image using non-maximal suppression.

There are several members in the CI family, such as CF (Coin Filter), IF (Iris Filter), ARF (Adaptive Ring Filter) and the SBF [24, 25]. The differences in these members are the definition of the support region *R*. The SBF filter has a band with a fixed width support region, the average convergence index in band width can be maximized by changing the position of the band in each radius direction. Figure 7(a) depicts the scheme of the support region in SBF. The definitions of the SBF filter is as follow:

3

With


where *Grad*_*nC*_ and *Grad*_*nR*_ are the column and row gradients at image position n, respectively, *P*_*n*_ is the number of support region lines irradiated from the center pixel (*x*, *y*), *Bw* is the sliding band width, *r* is the position of the band center in the support region line ranging from *R*_min_ to *R*_max_, and cos(*φ*_*rad*_ − *α*(*φ*_*rad*_, *n*)) is the angle between the gradient vector at (*φ*_*rad*_, *n*) and the direction of *φ*_*rad*_.

The SBF was employed to develop a cell segmentation method in Quelhas’ work [15], but it detects only convergence or divergence in the dark field. However, the cell membrane in the bright field is distinct from the background with the location of both convergence and divergence, see Figure 7(c) and (d), and cytoplasm is not visible. This leads to the *Conv*(*rad*, *n*) in SBF filter does not fit the bright field cell area. By ignoring the affection of divergence and convergence in the bright field, we transform the convergence index in SBF to facilitate it to fit the cell membrane area well. In this paper, the Transformed Sliding Band Filter (TSBF) is given by:
4

with


where *ABS*(*CI*) is the absolute value of the convergence index at pixel (*rad*, *n*), this eliminates the characteristic of divergence and convergence at the edge of cells, *Grad*(*rad*, *n*) is the gradient value at pixel (*rad*, *n*), *ω* is a weight parameter designed for the image variation on uneven illumination and out of focus (we set *ω* = 1 in this paper as the default value). After applying the TSBF filter to insect cells, filter output results indicate that the cell centers are associated with the locations of filter local maximal value.

The classical SBF filter was employed by the image processing community to enhance the edge and center in the rounded convex region. In this paper, we use transformed SBF, named TSBF to detect cells in the bright field. The given insect cell image, as shown in Figure 7(b), was processed by our designed TSBF filter, and the results show that the center of cell was enhanced by this method, as shown in Figure 7(c). Furthermore, the enhanced center was assumed as the center of each cell.

A local Non-Maximum Suppression (NMS) was applied to detect the local maximal value which was the assumed cell center, details of the NMS were listed in Algorithm 1, where W and H represent the width and height of a given image, and position (k, l) and (k_1_, l_1_) are pixels in the (n + 1) × (n + 1) region [26, 27]. In this part, the NMS can be formulated as the local maximum search, where one local maximum, excluding itself is greater than all its neighbors. 2D square images of (n + 1) × (n + 1) centered on the pixel were under consideration in this paper.

### Cell detector evaluation

The proposed TSBF method for detecting cells in a bright field was tested on the insect cell image datasets collected using the protocol of BEVS. To evaluate the accuracy of the proposed method, an error rate estimation process was employed. We used manual counting results as the GT (Ground Truth), and then the performance of the proposed cell detector was evaluated by the error criterion according to equation five and equation six.
56

with


where Error Rate of TSBF (ERT) is the relative error rate at a certain cell density calculated using the TSBF filter, Total Error Rate of TSBF (TERT) is the total error rate of all cell density calculated using TSBF, *NT* is the number of cell samples in different densities, *GT* is the Ground Truth, *Sample* _ *i* is cell density of a sample from a certain time counted by our lab collaborators. *N*_*si*_ is the average number of *Sample* _ *i*. For example, after recovering from the liquid nutrition, cell suspension with a density of 5 × 10^5^ cells/mL was made by an experienced lab collaborator as the starting point, 3 samples were collected each 24th hour until the 216th hour (the 9th day) and then counted by 3 lab collaborators and the proposed method.

## Results and discussion

We assume that the cell diameters in all images are about the same size, and that most parameters of our method are intuitive. For the insect cell image dataset, the following parameters were set based on direct visual inspections. For the cell detection, we evaluated the cell diameters by direct observations. We set *R*_min_ = 8 pixels, *R*_max_ = 30 pixels for the maximum and the minimal cell radius respectively; this is to make sure the support region of maximum radius covering the full cell. Regarding the remaining SBF filter parameters we adopt the default setting *N* = 32 [15], and *d* = 6, according to the width of the cell edge in the image by direct observations.

**Figure 8:**
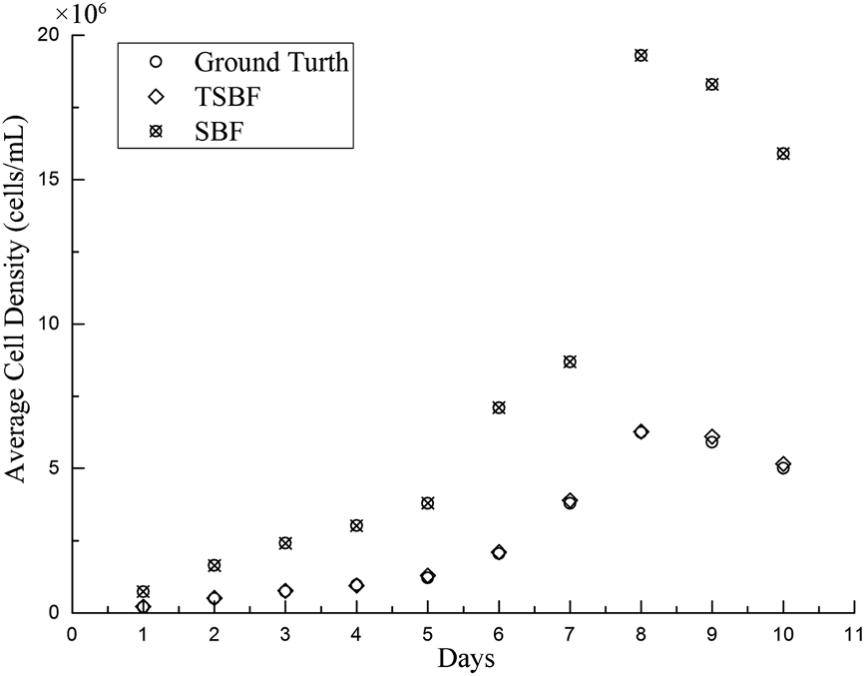
**Average cell counting results by 3 lab collaborators, the TSBF method and the SBF method during the cultivation period.**

The proposed method was implemented on a desktop computer with an Intel 1.86 GHz CPU and 2GB RAM memory. The proposed approach is currently fully implemented with MATLAB R2012a. Although the TSBF filter is a computationally intensive operation, our method currently takes approximately 22 mins to perform the cell detection of a 512 × 512 pixel image and it will be improved to perform in less time by using GPUs in the near future.Cell counting is a complicated and tedious task in many cell-based experiments and usually performed manually by microscopic observations. This leads to many problems such as labor intensity, time consumption, and proneness to errors intro-and inter-persons. Thus, effective cell detectors are needed for biotechnicians to master this task during cell based experiments. Being an excellent cell detector in bright fields, it must satisfy some criteria such as providing the accuracy and reliability of manual counting results. In this paper, the introduced cell counting method in the bright field for insect cells can facilitate the BEVS’s manipulation and improve its efficiency. We test our method on confocal images of insect cells collected from different periods of cultivation. For each sample of insect cells, masks were employed to cover the A1 through A5 regions of the chambers separately in Figure 2, and then we manually calculated each sample by 3 lab collaborators to create the ground truth for investigating the variations among manual counting, the TSBF method and the SBF method.Counting insect cells is usually performed manually by chambers under microscopy. In addition, the results have to be repeated several times in the same view and then an average number is taken as the relative accurate number. Compared with the manual counting, the proposed method can obtain results with an intuitive parameter setting and the variance on cell density per mL ranging from 7,000 cells to 200,000 cells. We choose 3 samples each day to count the results by our method and manual counting during the cultivation period. The samples were collected from the cultivation medium by sequence and named sample 1, sample 2 and sample 3. Figure 8 compared the average density of cells between manual, TSBF, and SBF methods during the cultivation period. After applying the TSBF method to the cell counting task, there is still a variance in counting results up to 200,000 cells per milliliter. Although there is an enormous variation between these methods, it can be eliminated during the huge cultivation of insect cells after evaluation by an experienced biotechnologist.

**Figure 9:**
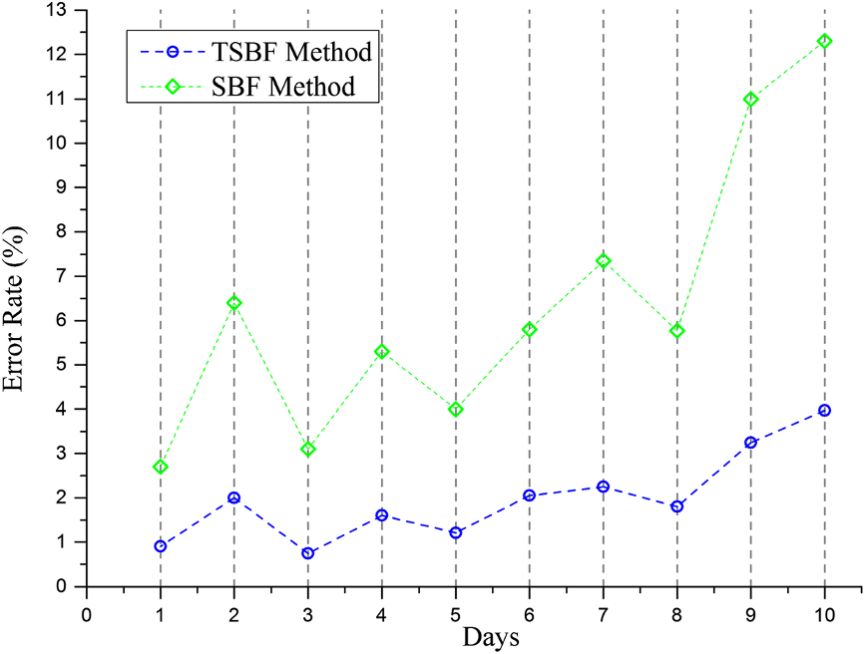
**Error rate evaluation of the TSBF method and SBF method.**

**Figure 10:**
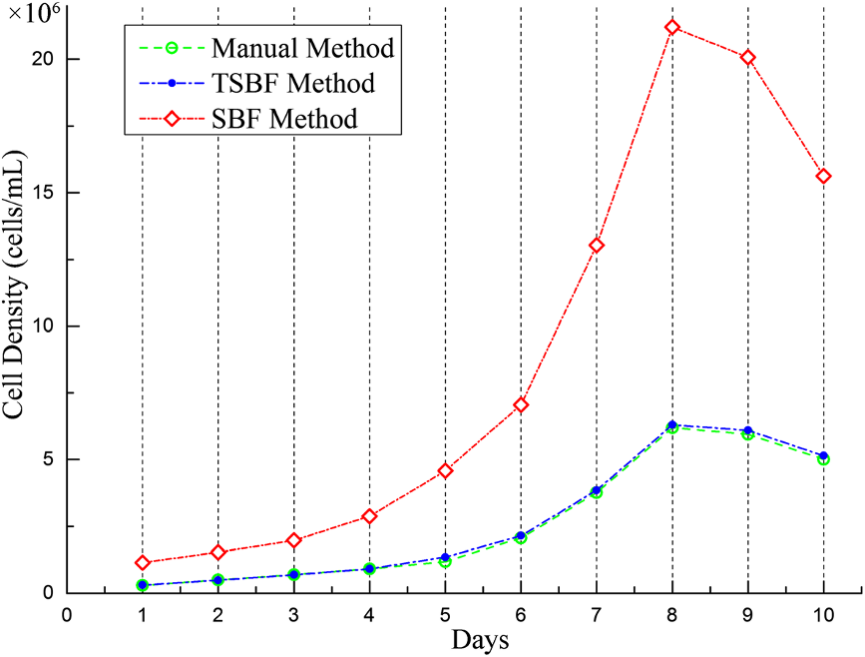
**Growth curve comparison results between the Ground truth, TSBF method and SBF method.**

**Figure 11:**
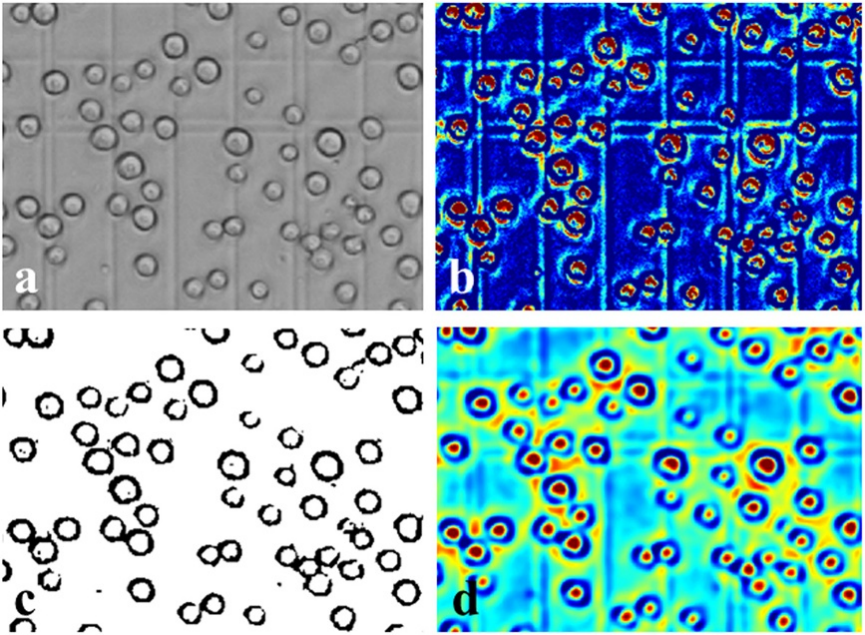
***Sf9*** **cell detection comparison between Laplacian of Gaussian (LoG) method (a), intensity threshold method (b), TSBF method (c), and (d) was a magnification of parts in (c).**

The error rate is an important criterion to evaluate the accuracy of a cell detector. The average error rate of our TSBF method is 2.21% on average, ranging from 0.89% to 3.97% as shown in Figure 9. As an estimated value, the ideal cell density in the log phase for experiments is 5.5 × 10^6^. As an experimental experience in cell cultural, this density is permitted during 5.3 × 10^6^ ~ 5.7 × 10^6^, and the difference is higher than 2%, so this error rate can be eliminated in cell counting. In our research, the insect cells were cultivated using the suspension method and they usually crowded together when they were out of the shaker. Some of the cells still exhibited crowding after being injected into the chamber, and these cells were usually identified as one cell by manual counting but were detected as 2, 3 or more cells by the TSBF method. This may lead to increase the total error rate as insect cells grow. To verify this hypothesis, we diluted the cell suspension to make sure cells were separated from each other at the 4th and 8th day, and the results exhibited a decrease in error rate. But after cells grew into the stationary phase, some cells were corrupted for lacking nutrition, and the TSBF method detected the bigger cell corruption as a single cell, which leads to an increase in the error rate. Fortunately, insect cells in this phase were out of use for experiments. So, this defect can be omitted.The growth curve can reflect the insect cells growth manner. Drawing an accurate growth curve in the pre-culture stage can enable bio-technologists to decide when to seed baculovirus into insect cells and when to perform follow-up experiments. We compared the TSBF method and manual counting method on the preparation of the growth curve. Figure 10 shows the growth curve where the solid green line and dotted blue line correspond to the manual and TSBF methods, respectively. The two curves match well before and during the exponential growth phase with little separation. But, they do not match so well after the stationary phase. The reason for this is that the TSBF method detects the cell corruptions as a single cell and therefore leads to a detected cell density larger than the real density. Fortunately, the insect cells in this period were in the decline phase and the cells were out of use which has no effect on follow-up experiments, so the negative effect can be eliminated.Cell counting is an important process in cell based experiments. Although various methods and software have been developed to aid biotechnologists in performing this task, most of the methods are limited in their application scopes and cannot be applied to other kinds of cells or cell lines. The TSBF method can be applied to most cells like insect cells in bright field microscopy to perform the cell counting task. Comparison results between the TSBF and traditional image enhancement methods demonstrated an excellent performance of our proposed method in insect cell image enhancement. Figure 10 shows the comparisons between Laplacian of Gaussian (LOG), image threshold and the TSBF method. Results show that the TSBF method proposed in this paper can detect the insect cells with a higher filter resonance value in the center of the cell. Comparing with the other two traditionally methods, only the LOG method can detect the single cell in the image, but the background noise heavily affects the filter response and the detecting result was not as good as the TSBF method. The image threshold method merely detects the cell membrane area in the bright field images. More details were shown in Figure 11(a) to (d). In addition, the TSBF method can also eliminate the background noise in the image, as it is depicted in Figure 11(c).

Additionally, as a common scene for biological experimental workers, most of cells cultivated in vitro usually exhibited the same morphology in bright field, and thus, all of these cell lines’ counting task which needs Hemacytometer can be solved by our proposed method.

## Conclusion

In contrast to traditional methods, in this paper we presented a semi-automated cell counting protocol for BEVS in bright field microscopy image datasets. Comparing with the SBF filter, a transformed non-linear filter (TSBF) was employed to detect the insect cells in the protocol according to the cells gradient vector distribution manner. Since all four of the cell lines exhibited the same gradient vector distribution in the bright field, we tested on the Sf9 insect cell rather than all the 4 cell lines. The proposed method is adaptive and efficient.

To test the accuracy of the proposed cell counting method, we evaluated the error rate. In experiments, the TSBF method showed an excellent performance with an average error rate of 2.21%, ranging from 0.89% to 3.97%, compared with human manual counting by 3 lab collaborators. Also, the error rate of 2.21% can be eliminated in cell counting during cell cultivation. In addition, growth curve evaluation indicates that the TSBF method can be applied to the protocol of BEVS for its host cell density evaluation and growth stage identification.

The ultimate goal of this study is to help biotechnologists count the host cells of baculovirus in the BEVS protocol. As demonstrated in our experiments, the proposed method is very accurate compared with manual counting. Therefore, the application of our proposed bright field cell counting method can clearly benefit the protocol of BEVS. Finally, as a non-linear filter, the TSBF method is computationally expensive. Thus, our future work is aiming to improve the method so it runs in less time by using GPUs.

Part of this work was introduced in a conference paper of EMBC 2012 [28].
